# A commentary on “Long-term outcomes of single-incision laparoscopic surgery for colorectal cancer: a single-center, open label, randomized controlled trial”

**DOI:** 10.1097/JS9.0000000000003057

**Published:** 2025-07-18

**Authors:** Jiali Huang, Jie Lin

**Affiliations:** a Shenzhen Hospital (Futian) of Guangzhou University of Chinese Medicine, Shenzhen, Guangdong, China; b Guangzhou University of Chinese Medicine, Guangzhou, Guangdong, China

*Dear Editor,*

We read with great interest the recent randomized controlled trial by Dr. Song and colleagues^[1,2]^, which provides valuable insights into the long-term outcomes of single-incision laparoscopic surgery (SILS) versus conventional laparoscopic surgery (CLS) for colorectal cancer. Their study offers meaningful evidence by meticulously reporting 5-year disease-free survival (DFS) and overall survival (OS) outcomes over a median follow-up of 71.3 months, thus helping to clarify the role of SILS in modern colorectal cancer management. While this ambitious effort contributes significantly to the ongoing dialogue on minimally invasive surgical strategies and oncological safety benchmarks, several aspects warrant further discussion regarding the statistical rigor and interpretability of the reported findings.

Firstly, the challenge of multiple subgroup analyses without adequate adjustment for multiplicity. A pivotal strength of this trial is the attempt to probe the consistency of treatment effects across a diverse spectrum of baseline characteristics. Specifically, the authors performed subgroup analyses for 5-year DFS across strata including age, sex, body mass index (BMI), tumor stage, tumor location, tumor size, and ECOG performance status (Figure 4). While no statistically significant heterogeneity was observed among subgroups, the authors did not implement corrections for the increased risk of Type I error intrinsic to multiple hypothesis testing. As the number of subgroup comparisons increases, so does the likelihood of finding a “statistically significant” effect purely by chance a well-known pitfall in clinical research[3]. Figure 4 reports hazard ratios and confidence intervals for each subgroup, yet the associated p-values are not adjusted for multiple comparisons using methods such as Bonferroni or the false discovery rate (FDR). This omission raises the specter of over-interpreting nominal findings, especially when the sample size within each stratum is small, as visually indicated by the narrow confidence bars in the forest plot. Moreover, interaction p-values are not reported, making it impossible to formally test whether the treatment effect differs between subgroups—a critical omission given the recognized importance of interaction testing in the robust interpretation of subgroup effects[4]. Recent guidelines and consensus papers recommend prespecifying a limited set of clinically justified subgroup analyses and adjusting for multiplicity, especially in non-inferiority designs where overinterpretation of subgroup findings can distort the primary trial conclusions[5]. Without such safeguards, the trial’s interpretive bandwidth may be compromised by spurious associations.

Secondly, the core issue of event scarcity and limited statistical power. Perhaps the most consequential limitation of this otherwise well-conducted trial lies in the surprisingly low number of endpoint events. As detailed in Table 3, only 23 out of 193 patients (11 in SILS, 12 in CLS) experienced recurrence or metastasis over the 5-year follow-up period, while just 22 deaths were recorded (11 in each group). This translates to an event rate of merely 12% for recurrence and 11% for mortality. With so few failures driving the event-driven endpoints, the resulting hazard ratios for DFS (HR 1.03, 95% CI 0.47–2.21, *P* = 0.95) and OS (HR 1.26, 95% CI 0.52–3.02, *P* = 0.61) are encumbered by wide confidence intervals that span unity, signaling a lack of statistical precision and increasing the risk of a Type II error. Notably, this statistical fragility is further underscored in the subgroup analysis (Figure 4), where event counts are so sparse that most stratum-specific effect estimates are highly unstable. For time-to-event endpoints like DFS and OS, a sufficient number of outcome events is essential to ensure adequate statistical power and to support reliable inferences[6]. The current study’s findings of non-inferiority between SILS and CLS could thus reflect an underpowered design rather than genuine therapeutic equivalence. Given the importance of accurately quantifying potential differences in oncological safety, future studies should prioritize event-driven design with adequate sample sizes or lengthier follow-up to achieve a robust number of failures.

The issue is not merely technical: underpowered studies risk missing true differences in efficacy or safety, and their confidence intervals may be so broad as to encompass both clinically meaningful benefit and harm. For example, the hazard ratio for DFS in this study (HR 1.03) ranges from a 53% risk reduction to a 121% increased risk—an interpretive spread that should caution against firm clinical recommendations without further evidence.

In conclusion, while Dr. Song and colleagues have charted an impressive and rigorous course toward advancing the science of minimally invasive colorectal surgery, we urge caution in the interpretation of their results. Their trial illuminates the enormous potential of SILS but also underscores the necessity for statistical rigor in both subgroup analyses and event-driven outcome research.

## Data Availability

No datasets were generated or analyzed during the current study.

## Research registration unique identifying number (UIN)

Not applicable for this Correspondence.
